# Self-powered and self-calibrated sensing system for real-time environmental monitoring

**DOI:** 10.1126/sciadv.adw3745

**Published:** 2025-06-11

**Authors:** Jun Ma, Hao Sun, Zhekai Chu, Jiling Zhao, Zhiju Yang, Shuhai Liu, Juan Wen, Yong Qin

**Affiliations:** ^1^ Institute of Nanoscience and Nanotechnology, School of Materials and Energy, Lanzhou University, Lanzhou 730000, Gansu, China.; ^2^ MIIT Key Laboratory of Complex-field Intelligent Exploration, Beijing Institute of Technology, Beijing 100081, China.

## Abstract

Self-powered sensing related to triboelectric nanogenerator (TENG) as a sustainable self-sufficient power source for environmental monitoring by harvesting energy from a living environment is critical in the field of energy and environmental science. However, features of small energy-density and irregularity of environmental energy make it difficult to use directly for real-time environmental monitoring. Here, we report a self-powered and self-calibrated environmental monitoring system (SSEMS) composed of TENG, a calibration resistor, and a sensor network in parallel for real-time temperature and humidity monitoring. The calibration resistor can monitor in real time the irregular output of TENG from the irregularity of rainfalls. SSEMS uses this calibrating signal to calibrate the sensing signal in real time, achieving accurate sensing with an error margin less than 5.0%. We applied SSEMS under waterfalls and rainfalls to monitor in real time the environmental temperature and relative humidity with a sensing error as low as 1.0%. This work promotes self-powering technology one step further to practical applications.

## Teaser

A self-powered and self-calibrated environmental monitoring system is achieved for real-time temperature and humidity monitoring.

## INTRODUCTION

Environmental monitoring plays an increasingly crucial and indispensable role in natural ecosystem protection, disaster warning, battlefield detection, and many more (*1*–*3*). In a typical environmental monitoring system (EMS) (*4*), various sensors are integrated to monitor temperature, humidity, wind speed, air quality, and so on. The current powering technology for these sensors mainly relies on rechargeable batteries. But for the near future, with the number and density of EMS widely distributed in the environment that could be large, replacing batteries for these large numbers of EMS is becoming challenging and even impractical especially for those conditions where humans and machines are difficult to reach (*5*). For this reason, seeking of power sources for driving various sensors (sensor network) and achieving continuous environmental monitoring are becoming increasingly important.

In recent years, due to triboelectricity (*6*, *7*), piezoelectricity (*8*, *9*), thermoelectricity (*10*, *11*), photoelectricity (*12*, *13*), magnetoelectricity (*14*), and pyroelectricity (*15*, *16*), environmental energy harvesting that scavenges solar (*17*), wind (*18*), and wave energy (*19*) from the natural environment provides a possibility to address the powering challenge for driving sensors. Because of collecting energy from the environment without external power source, the system based on this energy-harvesting technology can be a sustainable self-sufficient system [self-powered system; (*20*, *21*)] with strong environmental adaptability and energy sustainability. Up to now, the one based on triboelectric nanogenerator (TENG) that can directly harvest mechanical energy from living environments like natural rainfalls is becoming increasingly important in the field of EMS and attracts more and more attention worldwide (*22*–*24*).

Because most of the mechanical energy in the environment such as natural rainfall generally has the features of low energy density and extreme irregularity, this makes the electricity generated per cycle by TENG very small and exhibits high irregularity in the amplitude, frequency, and density. Directly using this irregular electricity as power source for EMS will lead to an irregular loop current fluctuation in EMS, which will submerge the sensing signal (loop current change caused by environmental stimulus), resulting in poor sensing performance of EMS. So, a complex power management composed of ac/dc converter, energy extraction circuit, storage cell (battery or capacitor), and voltage regulator (i.e., LTC3588) (*25*, *26*) is developed to collect the irregular output of TENG and then stably power the sensors at intervals. Although this additional power management converts the irregular output of TENG into a stable power source, thereby avoiding the influence of irregular output of TENG on the sensing signal of EMS, it leads to additional energy loss and makes EMS only work intermittently, like “sleep” to “wake” working mode (*27*), which severely limits the ability of EMS for real-time environmental monitoring. So, it highly desirable to find schemes for developing a self-powered sensing system for real-time environmental monitoring.

In this work, we proposed and developed a self-powered and self-calibrated environmental monitoring system (SSEMS) based on a typical raindrop-TENG (R-TENG), which can sense and harvest the mechanical energy of raindrops widely existing in nature for real-time monitoring. This SSEMS is realized by using a shunt circuit to record in real time the irregular output of R-TENG and thereby calibrate the sensing signal from the irregular output of R-TENG. Our results show that SSEMS can monitor in real time the environmental temperature and humidity with errors as low as 1% by collecting the mechanical energy in the environment, which is as good in performance as those sensors being powered by a commercial constant voltage source. This work provides a simple but effective method to address the challenge of real-time environmental monitoring and is expected to promote self-powering technology one step further in practical applications.

## RESULTS

### Design and working mechanism of SSEMS

**Fig. 1.**
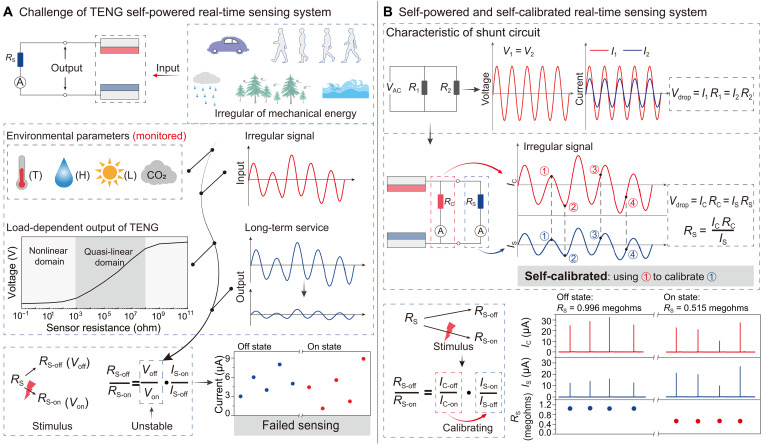
Design and mechanism of SSEMS. (**A**) Current challenge of self-powered real-time monitoring system based on TENG. Various factors lead to failed sensing of self-powered real-time monitoring system. T, temperature; H, humidity; L, light. (**B**) Design and working mechanism of SSEMS to solve the challenge.

To achieve real-time environmental monitoring, a self-powered EMS (SEMS) is traditionally constructed by directly connecting sensors (particularly, resistive sensors) with TENG and is directly powered by the TENG as shown in Fig. 1A. But for this SEMS, there are the following three issues that will lead to a decrease in the sensing accuracy of the sensing system. First, the irregularity of mechanical energy in complex environment results in irregular output of TENG. Second, the degradation of TENG or its charging state results in a degraded output. Third, the typical load-dependent output characteristic of TENG makes it inevitable that its output also fluctuates with the sensor resistance, which varies with the stimuli (to be sensed). Above three issues alone or together will lead to the TENG’s output voltage [*V*_out_ (*R*), a function of load resistance *R*] itself different before [*V*_off_ = *V*_out_ (*R*_S−off_)] and after [*V*_on_ = *V*_out_ (*R*_S−on_)] the sensor is stimulated by environmental factors such as light, force, or temperature. As a result, the on/off ratio obtained by SEMS will be expressed as

$$\begin{array}{c} \text{on}/{\text{off ratio}}_{\text{SEMS}}=\frac{I_{S-\text{on}}}{I_{s-\text{off}}}=\frac{V_{\text{on}}/R_{S-\text{on}}}{V_{\text{off}}/R_{S-\text{off}}}\\ \;=\frac{V_{\text{on}}}{V_{\text{off}}}\cdot \frac{R_{S-\text{off}}}{R_{S-\text{on}}}\neq \frac{R_{S-\text{off}}}{R_{S-\text{on}}}=\text{on}/{\text{off ratio}}_{\text{actual}} \end{array}$$ (1)

where *R*_S−off_ and *R*_S−on_ represent the sensor resistance before and after being stimulated, respectively, and their corresponding current is *I*_S−off_ and *I*_S−on_, respectively. Because *V*_off_ is not equal to *V*_on_, the obtained on/off ratio by SEMS is not equal to the actual one (*R*_S−off_/*R*_S−on_), resulting in failed sensing (lower of Fig. 1A).

Considering the case of shunt circuits with two resistors *R*_1_ and *R*_2_ in parallel as shown in Fig. 1B, the voltage drops *V*_drop_ across these two resistors are equal to each other, and their currents *I*_1_ and *I*_2_ follow Ohm’s law

$$V_{\text{drop}}=I_{1}R_{1}=I_{2}R_{2}$$ (2)

This equal *V*_drop_ of shunt circuits in parallel inspires us to apply the *V*_drop_ of one shunt circuit to calibrate the one of another circuit. Particularly, we use a calibration resistor *R*_C_ (a constant resistance as *R*_1_ in Eq. 2) to calibrate the sensor resistor *R*_S_ (as *R*_2_ in Eq. 2). By measuring the current in real time *I*_C_ (*I*_C–off_ and *I*_C–on_, respectively, for the current before and after the sensor stimulated) flowing through *R*_C_, we can record in real time the *V*_drop_ (=*I*_C_·*R*_C_) across *R*_C_ and *R*_S_, and so as to calibrate the irregular output of TENG. This process can be expressed by the following Eqs.

$$V_{\text{off}}=I_{C-\text{off}}\cdot R_{C}=I_{S-\text{off}}\cdot R_{S-\text{off}}$$ (3A)

$$V_{\text{on}}=I_{C-\text{on}}\cdot R_{C}=I_{S-\text{on}}\cdot R_{S-\text{on}}$$ (3B)

As can be seen, by introducing the shunt circuit of *R*_C_, we calibrated *V*_off_ and *V*_on_ in real time. In addition, as a result, we are able to capture in real time *R*_S−off_ and *R*_S−on_ (regarded as the sensing signal of the sensor) as Eqs. 4A and 4B and obtain the actual on/off ratio as Eq. 5

$$R_{S-\text{off}}=\frac{I_{C-\text{off}}\cdot R_{C}}{I_{S-\text{off}}}$$ (4A)

$$R_{S-\text{on}}=\frac{I_{C-\text{on}}\cdot R_{C}}{I_{S-\text{on}}}$$ (4B)

$$\begin{array}{c} \text{on}/{\text{off ratio}}_{\text{actual}}=\frac{R_{S-\text{off}}}{R_{S-\text{on}}}=\frac{\left(I_{C-\text{off}}\cdot R_{C}\right)/I_{S-\text{off}}}{\left(I_{C-\text{on}}\cdot R_{C}\right)/I_{S-\text{on}}}=\frac{I_{C-\text{off}}}{I_{C-\text{on}}}\cdot \frac{I_{S-\text{on}}}{I_{S-\text{off}}} \end{array}$$ (5)

By comparing the difference between Eqs. 1 and 5, it can be found that the introduction of the shunt circuit of *R*_C_ for calibration allows us to add a calibration item *I*_C−off_/*I*_C−on_, so that the actual on/off ratio of the sensor can be accurately obtained. Because the calibration is also powered by TENG, we call the calibration process self-calibration. In addition, this constructed EMS here is named self-powered and self-calibrated environmental monitoring system (SSEMS). It should be noted that the above discussion is based on the fact that *R*_C_ is a fixed value, and, when *R*_C_ changes with temperature, the temperature-dependent *R*_C_ may affect the sensing accuracy particularly at high temperature environment (>50°C) (fig. S1).

To preliminary verify the feasibility of SSEMS, we used R-TENG as a power source to conduct a simple demonstration as shown in Fig. 1B, setting the sensor resistance to 0.996 megohms (imitating the sensor resistance in off state) and 0.515 megohms (imitating the sensor resistance in on state). Despite the irregular output current of the R-TENG, the obtained resistances of the sensor by Eqs. 4A and 4B highly match the preset values (Fig. 1B, bottom), demonstrating the successful working of SSEMS. Additionally, the influence of *R*_C_ values on the sensing performance of SSEMS was also investigated (fig. S2). Theoretically, as long as there is an appropriate partial voltage on *R*_C_, its resistance value does not affect the working of SSEMS. We tested various *R*_C_ and various initial resistance values of *R*_S_ with a preset on/off ratio of 10. The measured on/off ratios match the preset values, further proving the fact that *R*_C_ also does not affect the sensing performance of SSEMS (fig. S2). Considering the above results, we selected a resistor of 514.2 kilohms (note: other values can also be selected) as *R*_C_ for following experiments.

### Sensing performance of SSEMS

**Fig. 2.**
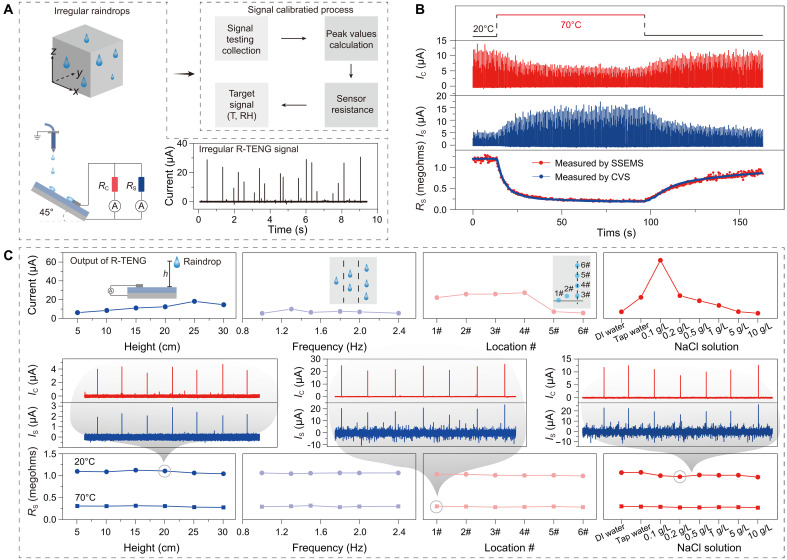
Sensing performance of SSEMS. (**A**) SSEMS based on R-TENG. Irregular raindrops lead to irregular output current of R-TENG, and a calibration process is needed to obtain the sensor resistance. T, temperature; RH, relative humidity. (**B**) Dynamic *R*_S_ (=*R*_C_·*I*_C_/*I*_S_) derived from *I*_S_ and calibrated by *I*_C_ over time in a practical test of SSEMS. CVS, constant voltage source. (**C**) Sensing performance of SSEMS insensitive to the output of R-TENG under various changing factors including raindrop height, frequency, location, and chemical composition. DI, deionized; L, liters.

To fully reveal the sensing performance of SSEMS, we chose R-TENG to construct SSEMS (Fig. 2A and fig. S3) (*28*). Raindrop is chosen as the energy source for SSEMS because of the following two reasons. First, raindrop is a natural carrier of mechanical energy widely distributed in our living environment, and its dynamic processes, such as impact and slide, provide great potential for self-powering systems. Second, the high irregularity of natural rainfalls in the volume, frequency, density, and location makes raindrop energy hard to be used for self-powering systems; if this kind of irregular mechanical energy can be used by SSEMS, then the high performance of SSEMS on irregular energy harvesting and accurate signal calibrating for sensing can be better proved.

The irregular raindrops lead to irregular output current of R-TENG, making the SSEMS unable to accurately sense the target signal only by *I*_C_ or *I*_S_. To use the irregular raindrop energy, a calibration process of “acquiring *I*_C_ and *I*_S_ waveforms,” “capturing peak values,” and “calculating sensor resistance” is needed to lastly obtain the target signal. As shown in Fig. 2B, we tested SSEMS with temperature switched between 20° and 70°C, simultaneously acquired the dynamic *I*_C_ and *I*_S_ waveforms, and captured their peak values over time. Using the above calibration process, we calculated *R*_S_ by the formula *R*_C_·*I*_C_/*I*_S_ at each time and lastly plotted its dynamic curve with temperature. As a comparison, SSEMS can accurately obtain the dynamic *R*_S_, which is highly consistent with the actual sensor resistance obtained by a constant voltage source. As a contrast, a self-powered sensing system without the calibration process has a huge noise due to the irregular output current of R-TENG (fig. S4).

To clarify the sensing performance of SSEMS not influenced by the output variation of R-TENG, we systematically studied the SSEMS in consideration of various factors including raindrop height, frequency, location, and chemical composition. As shown in Fig. 2C (top), the output current of R-TENG varies markedly with the changes of these factors, indicating that any one factor varication will cause the output of R-TENG to be irregular. By introducing the calibration process, SSEMS can accurately obtain the sensor resistances corresponding to different temperatures (Fig. 2C, bottom) and is totally insensitive to the output of R-TENG. In addition, we also verified that SSEMS powered by contact-separation mode TENG and different positions of TENG AC signals can be used as effective sampling points (fig. S5).

### High-precision temperature and RH sensing of SSEMS

**Fig. 3.**
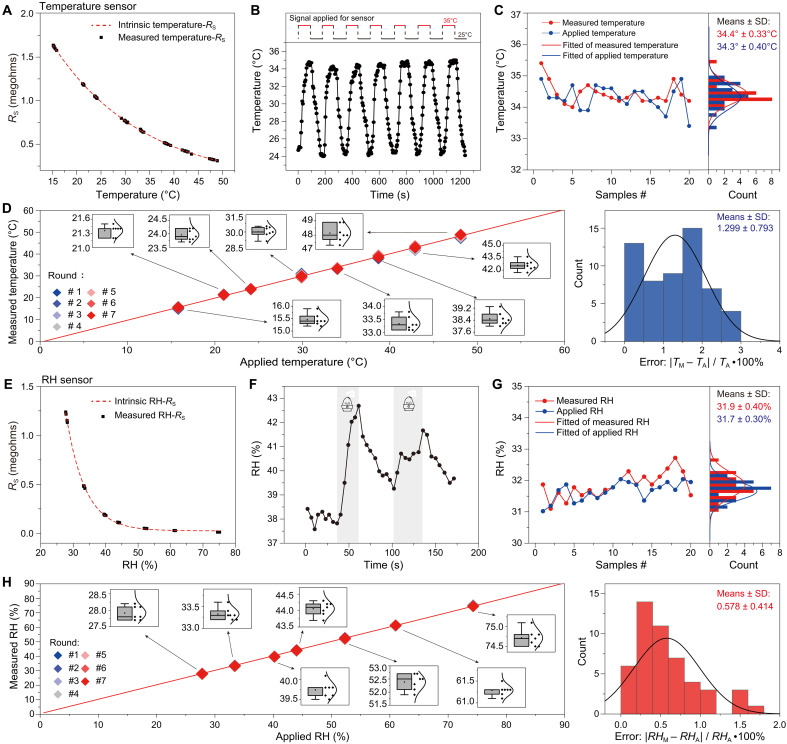
High-precision temperature and RH sensing of SSEMS. (**A**) Temperature-*R*_S_ characteristics of a commercial temperature sensor measured by SSEMS (black points) in comparison with the intrinsic characteristics (dotted line). (**B**) Dynamic temperature response measured by SSEMS under cyclic test ranged from 25° to 35°C. (**C**) Summary of static temperature sensing performances of 20 SSEMSs. (**D**) High-precision static temperature sensing of SSEMS at different temperature and its relative error. Insets: Distribution of seven data points measured at each temperature. *T*_M_, measured temperature; *T*_A_, applied temperature. (**E**) RH-*R*_S_ characteristics of a commercial RH sensor measured by SSEMS (black points) in comparison with the intrinsic characteristics (dashed line). (**F**) Dynamic RH response measured by SSEMS under a process of humidify (the gray background means the process of humidifying with a humidifier). (**G**) Summary of static RH sensing performances of 20 SSEMSs. (**H**) High-precision static RH sensing of SSEMS at different RH and its relative error. Insets: Distribution of seven data points measured at each RH. *RH*_M_, measured RH; *RH*_A_, applied RH.

Furthermore, we applied SSEMS for both temperature sensing and relative humidity (RH) sensing to evaluate its detection precision. As shown in Fig. 3A, an exponential relationship between *R*_S_ and temperature was measured by SSEMS, which is consistent with the intrinsic temperature-*R*_S_ characteristics of the commercial temperature sensor (fig. S6A). This consistency, in turn, reflects the correctness of sensing signal by SSEMS. On the basis of this, we carried out a dynamic temperature response measurement by SSEMS under cyclic test ranged from 25° to 35°C (Fig. 3B). It can be found that SSEMS can accurately detect the temperature changes in real time, having a measurement accuracy within ±0.9°C. The resistance change of temperature sensors is also shown in fig. S7A. To exclude the influence of individual commercial temperature sensors on our evaluation, we tested static self-powered sensing performances (sensing a fixed temperature) of 20 SSEMSs based on 20 commercial temperature sensors around 35°C and summarized their sensing performance in Fig. 3C. As can be seen, the measured temperature by SSEMSs closely matches the actual temperature, and the overall temperature obeys normal distribution with a relative error less than 2.5% (fig. S7B). In addition to 35°C, we also tested the SSEMS in a wider temperature range (15° to 50°C) in Fig. 3D. It can be found that there is a linear relationship between the measured temperature by SSEMS and the actual temperature. To ensure the reliability of evaluation, seven repeated measurements were conducted at each temperature, and their detailed distributions are presented in the inset of Fig. 3D. Upon further analysis, the detection error (relative error) is less than 3.0%, which is in consistent with that concluded from the measurement in Fig. 3C.

In addition to temperature sensing, RH sensing is another important content of environmental monitoring, so we evaluated the detection precision of SSEMS as self-powered RH sensing. Similar to temperature sensing (Fig. 3A), an exponential relationship between *R*_S_ and RH measured by SSEMS (Fig. 3E) is also consistent with the intrinsic RH-*R*_S_ characteristics of the commercial RH sensor (fig. S6B). At the same time, a dynamic RH response measured by the SSEMS during a humidification process is plotted in Fig. 3F, and the corresponding resistance change of the RH sensor is shown in fig. S7C. Due to our difficulty on dynamic control of RH, although the measured dynamic RH response curve seems not as good as that of temperature (Fig. 3B), the measured RH by SSEMS can reflect the environmental RH. Actually, the measured RH by SSEMS is consistent with that detected by a commercial hygrometer. As shown in Fig. 3G, 20 SSEMSs based on 20 commercial RH sensors can monitor the environmental RH, with a relative error less than 3.5% (fig. S7D). Moreover, SSEMS also exhibits high-precision RH sensing in a wider RH range (25 to ~75%) (Fig. 3H). Further analysis shows that the overall relative error of RH sensing is as low as 2.0%. These two evaluations regarding temperature sensing (Fig. 3, A to D) and RH sensing (Fig. 3, E to H) indicate the high detection precision of SSEMS with overall relative errors around 3.0%. It is important to mention that there is a big limitation of SSEMS, which can only work under rainy conditions. A possible solution to address this limitation is to combine the self-calibrated strategy with the complementary energy harvesting (energy of raindrop, wind, solar, etc.), and we verified the effectiveness of this approach (figs. S8 to S11).

### SSEMS based on R-TENG array

**Fig. 4.**
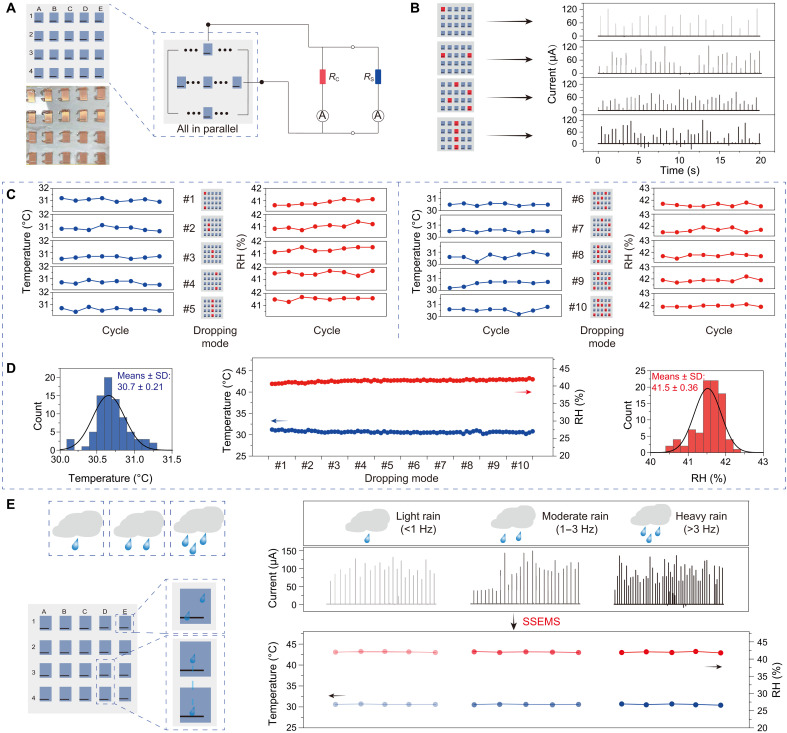
SSEMS based on R-TENG array. (**A**) Schematic diagram of SSEMS based on R-TENG array. (**B**) Output currents of R-TENG array under four different dropping modes. (**C**) Measured temperature and RH by SSEMS based on R-TENG array under different dropping modes. (**D**) Summary of measured temperature and RH from (C). (**E**) Temperature and RH sensing performance of SSEMS based on R-TENG array under light rain, moderate rain, and heavy rain.

In the calibration process, one output pulse of R-TENG means one powering and then one sampling; in other words, high-density output pulse can realize high sampling rate. To enhance the sampling rate and thus to achieve higher real-time measurement accuracy (positively correlated with the number of samplings per unit time) of SSEMS, we constructed SSEMS based on 4 × 5 R-TENG array, as illustrated in Fig. 4A. All the R-TENGs in the array are connected in parallel with each other. Each raindrop affecting the array, either by direct affecting or by sliding between R-TENGs, will trigger a sampling in the SSEMS. When multiple raindrops affect different R-TENGs during a short time, the SSEMS will perform a corresponding number of samplings, in other words, obtain a high sampling rate. As shown in Fig. 4B, when multiple raindrops fall on the array, the density of output pulse generated by the array will increase obviously, which makes the sampling rate of SSEMS increase greatly.

To further check whether this array composed of multiple R-TENGs will affect the sensing performance of SSEMS, we did a series of comparative experiments. As shown in Fig. 4C, we carried out temperature and RH measurements by SSEMS based on R-TENG array under different dropping modes (referring to the locations where raindrops affect the R-TENG array, which include drops falling on different parts of the array or drops affecting multiple adjacent R-TENG units) and found that the measured signals (temperature and RH) are highly consistent with each other and match the values measured by commercial temperature and RH sensors powered by batteries. Figure 4D summarizes the measured signal and analyzes the relative errors. The measured temperature and RH show a high stability under 10 dropping modes, and their average values were 31.7°C and 41.5% with relative errors less than 2.5 and 2.0%, respectively (fig. S12, A and B). These relative errors by multiple R-TENGs are smaller than that (3% for temperature sensing and 3.5% for RH sensing) by single R-TENG. This result proves that SSEMS based on R-TENG array has high-precision sensing performance comparable to the one based on single R-TENG.

As a demonstration in real condition, we explored the sensing performance of SSEMS based on R-TENG array under a simulated raindrop condition of light rain (<1 Hz), moderate rain (1 to 3 Hz), and heavy rain (>3 Hz). As shown in Fig. 4E, shower heads were used to simulate these raindrop conditions. Because the dropping position of raindrops on the array is highly irregular, the output current of R-TENG array is also irregular. Although the output current is irregular in all the three raindrop conditions, SSEMS based on R-TENG array is still able to accurately measure temperature and RH. Because the sampling rate is increased, the relative errors of measurement can be as low as 1% (fig. S12, C and D).

### Practical applications of SSEMS

**Fig. 5.**
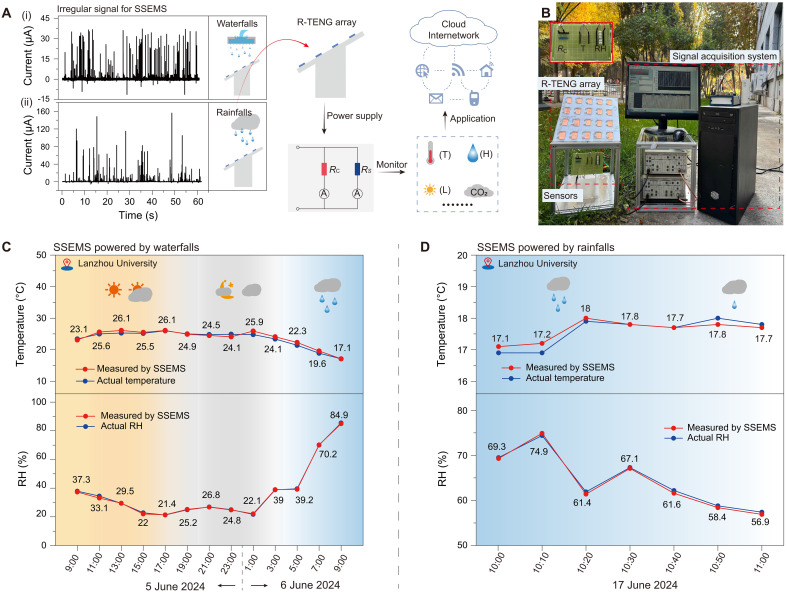
Practical applications of SSEMS under waterfalls and rainfalls for environmental temperature and RH monitoring. (**A**) Concept of SSEMS harvesting irregular energy from waterfalls and rainfalls to power sensors for environmental monitoring. T, temperature; H, humidity; L, light. (**B**) Photo image of SSEMS. Inset: The calibration resistor, and temperature and RH sensors. T, temperature; RH, relative humidity. (**C** and **D**) Temperature and RH recorded in real time by SSEMS under waterfalls (C) and rainfalls (D).

To further demonstrate the applications of SSEMS in real scenes, we respectively put SSEMS under waterfalls and rainfalls to record in real time the environmental temperature and RH at campus of Lanzhou University. Figure 5A shows the irregular output currents of R-TENG driven by waterfalls (i) and rainfalls (ii), respectively, and the scheme of SSEMS harvesting these two kinds of irregular energy from waterfalls and rainfalls to power sensors for environmental monitoring. Due to the previously discussed advantages of R-TENG array, here, we used R-TENG array as the power source of SSEMS (Fig. 5B).

Driven by waterfalls, SSEMS recorded in real time the temperature and the RH over time at the campus of Lanzhou University from 9:00 a.m. on 5 June 2024 to 9:00 a.m. on 6 June 2024 (Fig. 5C). As can be seen, the temperature remained stable but markedly decreased from 3:00 a.m. on 6 June, and was accompanied by an obvious increase in RH from 3:00 a.m. on 6 June. These recorded environmental parameters (temperature and RH) indicate a rainfall occurred at about 3:00 a.m. on 6 June, which is highly consistent with the actual data (blue points in Fig. 5C) and the actual weather on that day. Actually, the weather on that day is sunny from 9:00 a.m. to 3:00 p.m. on 5 June, cloudy from 3:00 p.m. to 1:00 a.m. on 6 June, and light rain to moderate rain from 1:00 a.m. to 9:00 a.m. on 6 June. It should be mentioned that the error between the recorded temperature and RH by SSEMS and the actual values (measured by commercial temperature and RH sensors powered by batteries) is less than 5%, and even as low as 1% (fig. S13, A and B), indicating that the SSEMS driven by waterfalls can accurately monitor environmental parameters.

Furthermore, we also put SSEMS under natural rainfalls to record in real time the temperature and the RH over time at the campus of Lanzhou University from 10:00 a.m. to 11:00 a.m. on 6 June 2024 (Fig. 5D). During this period, the rainfall gradually changed from moderate rain to light rain. As can be seen from Fig. 5D, when the rainfall gradually changed from moderate rain to light rain, the temperature slightly increased from 17.2° to 17.7°C, while the RH showed a downward trend from 69.3 to 56.9%. Through analyzing the recorded temperature and RH by SSEMS, we can distinguish the moderate rain from the light rain and reflect the weather change precisely. Moreover, the recorded temperature and RH are very close to the actual temperature and RH (measured by commercial temperature and RH sensors powered by batteries) (blue points in Fig. 5D), and the error is less than 1.8 and 1%, respectively, for temperature and RH monitoring (fig. S13, C and D). Above practical demonstrations show that the SSEMS can harvest irregular energy from waterfalls and rainfalls to power sensors for monitoring the typical environmental parameters temperature and RH in real time.

## DISCUSSION

In summary, a self-powered and self-calibrated sensing system for real-time environmental monitoring is proposed and developed. This system can power commercial sensors directly and achieve a highly accurate sensing response with an error margin less than 5.0%. In addition, for different sensors, this strategy can work successfully. By using waterfalls and raindrops, this system can monitor typical environmental parameters temperature and RH in real time, and the measurement error is as low as 1.0%. This work provides a universal approach to use irregular energy like droplets in the environment to precisely monitor environmental parameters, contributing to the practical applications of self-powered monitoring systems. It should be noted that self-calibrated strategy has potential in other fields while also having limitations. (i) Because the self-calibrated strategy is based on the principle of the shunt circuit, which means that the voltage applied to the different parallel sensors can be calibrated in real time by *R*_C_, achieving multiple environmental energies synergistically powering real-time multimode sensing is possible. (ii) However, due to the resistance of any material inevitably changing with temperature, making it unlikely to remain completely constant, the fluctuations in the calibration resistor contribute to an increase in the error of the sensing system. This is a very important problem and a limitation for our sensing system, and solving this problem is beneficial to the construction of a higher accuracy sensing system. (iii) Besides, the data acquisition of the sensing system requires additional power, which remains one of the biggest challenges for the self-powered sensing system and will be an important research direction in the future.

## MATERIALS AND METHODS

### Fabrication of TENG

First, a piece of polytetrafluoroethylene (PTFE) film with a thickness of 50 μm is cut into 3.0 cm by 2.0 cm and ultrasonically cleaned with acetone, ethanol, and deionized water for 15 min in sequence. After being dried by nitrogen, copper tape with a size of 2.8 cm by 1.8 cm was pasted on one side of the PTFE film as lower electrode. Second, a piece of cleaning Al film with a thickness of 20 μm cut into 1.5 cm by 0.2 cm was pasted with a piece of very thin double-sided tape on another side of the PTFE film as upper electrode.

### Commercial sensors

The commercial negative temperature coefficient temperature sensors (MF58) and RH sensors (CJHJ-31 W) were purchased from Yunyida Electronics Co. Ltd., Chuanju Electronics Co. Ltd., Zave Electronics Co. Ltd., respectively. The testing range of these temperature sensors is from −40° to 300°C, and its testing accuracy is 0.1°C. The testing range of these humidity sensors is 0 to 100%, and its testing accuracy is 0.1%. Figure S6 (A and B) gives the parameters of sensors.

### Electrical measurements

A peristaltic pump with adjustable speed was used to produce droplets at different speeds, and the volume of a droplet is 100 μl. The output voltage and output current were measured by a data acquisition card (National Instruments BNC-2120) together with a current amplifier (Stanford Research Systems Model SR570), and the sampling rate is 80,000 Hz. In the measurement of sensors, the droplet-TENGs or a commercial constant voltage source (Stanford Research Systems Model DS345) were used to power the sensors.
