# Supplementary material for: Self-powered and self-calibrated sensing system for real-time environmental monitoring
Source: Sci Adv. 2025 Jun 11;11(24):eadw3745. doi: 10.1126/sciadv.adw3745 (PMC12154185; doi:10.1126/sciadv.adw3745)
Supplement: Supplementary file 1 — Supplementary Text S1 to S4 Figs. S1 to S13 [file sciadv.adw3745_sm.pdf]

Science Advances
AAAS

Supplementary Materials for

Self-powered and self-calibrated sensing system for real-time environmental monitoring

Jun Ma *et al.*

Corresponding author: Shuhai Liu, liushuhai1991@live.cn; Juan Wen, wenj@lzu.edu.cn; Yong Qin, qinyong@lzu.edu.cn

Sci. Adv. **11**, eadw3745 (2025)
DOI: 10.1126/sciadv.adw3745

This PDF file includes:

Supplementary Text S1 to S4
Figs. S1 to S13

Supplementary Text 1. **Sensing accuracy of SSEMS affected by the temperature-dependent characteristic of R_C**

In order to study the relationship between SSEMS on/off ratio at different temperatures and their measurement standard deviation. We heated SSEMS from 20 °C to various temperatures ranging from 30 to 80 °C, with an enough heating time of 120 s. The results show that, as temperature increased, the on/off ratio increased from 1.35 to 4.42 (**fig. S1A**). At the same time, we observed that the standard deviation also gradually increased with temperature, rising from 0.05 at 30 °C to 0.24 at 80 °C (**fig. S1B**). This phenomenon indicates that higher temperatures affect the resistance value of R_C, leading to increased measurement standard deviation in the sensing system. This temperature-dependent accuracy shows that the temperature-dependent R_C needs to be considered when applying SSEMS in high-temperature environment (i.e., > 50 °C).

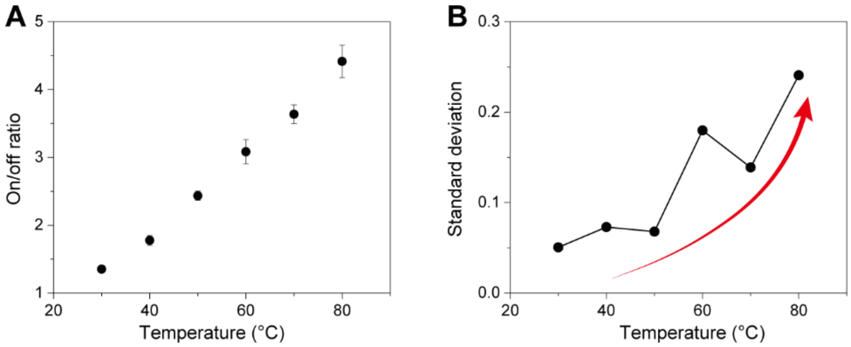

Fig. S1. On/off ratio of SSEMS under different temperature and the measurement standard deviation of SSEMS. (**A**) On/off ratio of SSEMS heated from 20 °C to various temperatures ranging from 30 to 80 °C (*N*=7). (**B**) The measurement standard deviation of SSEMS heated from 20 °C to various temperatures ranging from 30 to 80 °C.

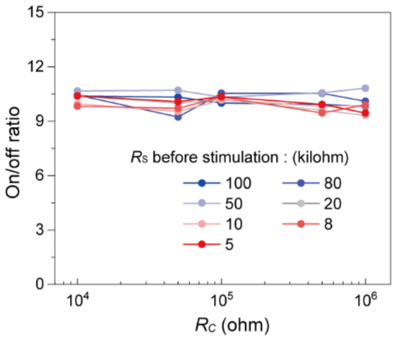

Fig. S2. Influence of R_C on the sensing performance of SSEMS. On/off ratio measured by SSEMS as a function of R_C. Different initial $R_{S\text{-off}}$ of 5 kilohms, 8 kilohms, 10 kilohms, 20 kilohms, 50 kilohms, 80 kilohms, and 100 kilohms, and a constant on/off ratio of 10 are pre-set.

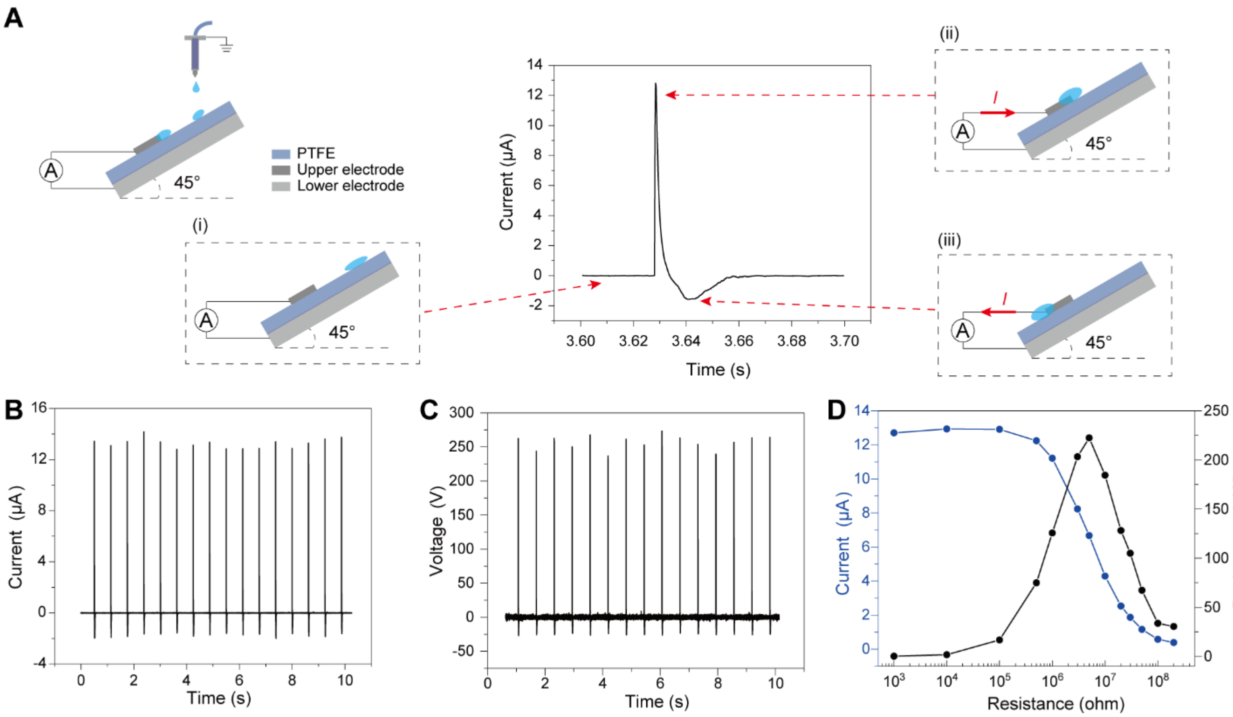

Fig. S3. Schematic diagram and output performance of R-TENG. (A) Schematic diagram of R-TENG. (**B**, **C**) Output current (B) and voltage (C) of R-TENG. **(D)** Current and peak power of R-TENG as a function of load.

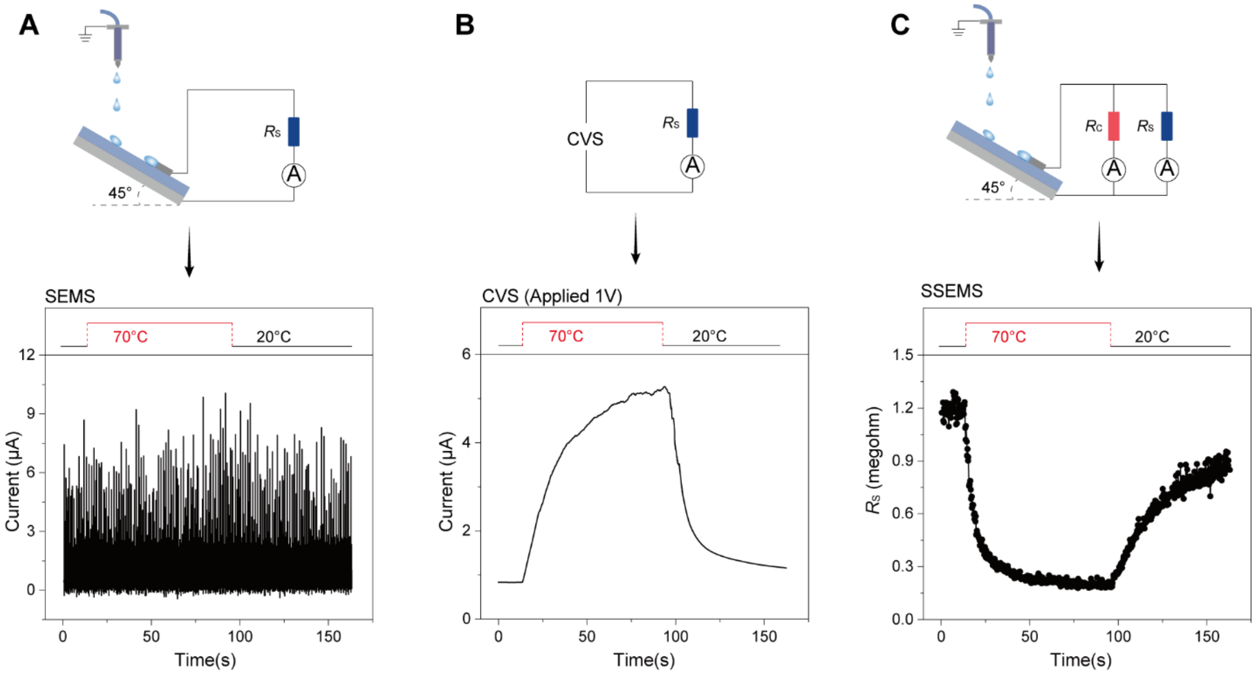

Fig. S4. Sensing performance of a temperature sensor powered by a R-TENG (with and without calibration process) and constant voltage source (CVS). The schematic and sensing performance of the temperature sensor powered by a R-TENG with (**A**) and without (**C**) calibration process and constant voltage source (CVS) (**B**).

Supplementary Text 2. **SSEMS powered by contact-separation mode TENG and effective sampling at different positions of AC signals**

In order to verify that AC signals can power SSEMS and prove that different positions of AC signals can be used as effective sampling points, we designed a contact-separation mode TENG (**fig. S5A**), where the friction layer achieves contact and separation under the knocking force, thus generating AC electrical signals. We built the SSEMS system shown in **fig. S5B** (fixed at 30 °C). In order to fully analyze the collected AC electrical signals, we selected different positions of current peaks for three independent samples (**fig. S5C**), and calculate the resistance value of the sensor based on a self-calibrated strategy. The experimental results show that no matter which peak position is sampled, as shown in **fig. S5D**, the calculated resistance value of the sensor remains the same (about 0.72 megohms). This result confirms two important conclusions: Firstly, AC signals can power SSEMS; Secondly, different positions on the AC signal can be used as effective sampling points, and the resistance value of the sensor can be accurately calculated, and which further expands the application potential of SSEMS under various signal conditions.

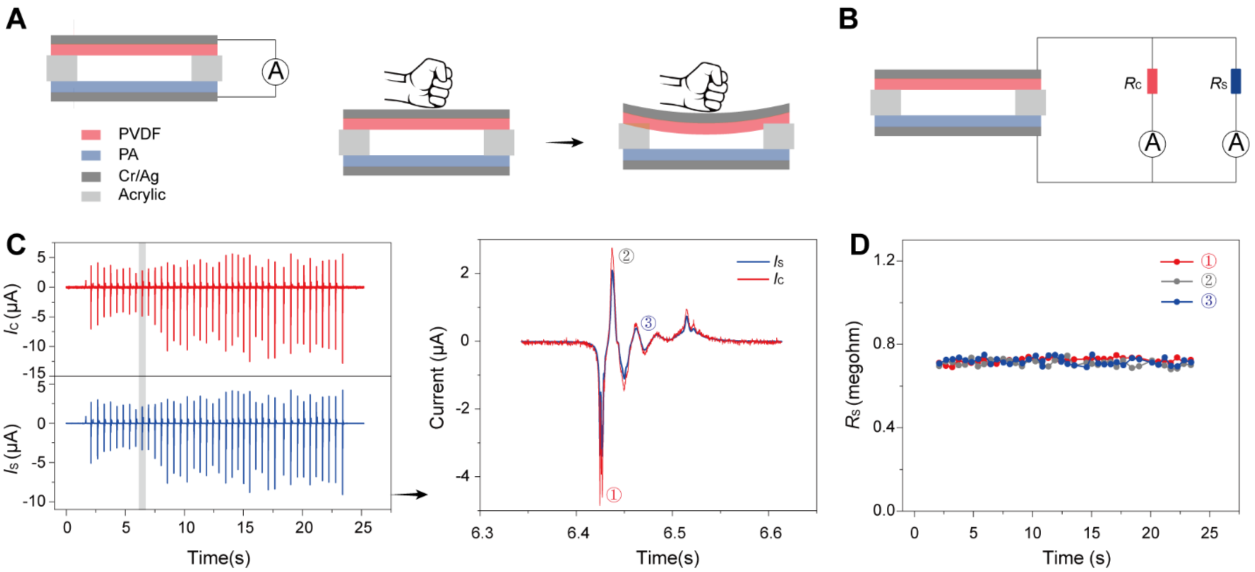

Fig. S5. Sensing performance of SSEMS powered by contact-separate TENG. (**A**) Schematic and work mechanism of contact-separate TENG. (**B**) Schematic of a SSEMS powered by contact-separate TENG. (**C**) The collected current of I_C and I_S, and local magnification of the current (**D**) Dynamic R_S ($=R_C \cdot I_C/I_S$) derived from I_S and calibrated by I_C over time in a practical test of SSEMS.

Supplementary Text 3. **Resistance-based sensor resistance and the monitored quantity should be measured in advance.**

Each new resistance-based sensor needs to be measured in advance to determine the correspondence between the sensor resistance and the monitored quantity, and this initial measurement is essential for achieving high accuracy SSEMS. The essence of our proposed self-calibration lies in achieving real-time determination of the voltage applied to the sensor by introducing a calibration resistor R_C, as shown in equation (S1) and (S2):

$$V_{\text{off}} = I_{\text{C-off}} \cdot R_{\text{C}} = I_{\text{S-off}} \cdot R_{\text{S-off}}, \tag{S1}$$

$$V_{\text{on}} = I_{\text{C-on}} \cdot R_{\text{C}} = I_{\text{S-on}} \cdot R_{\text{S-on}}. \tag{S2}$$

This enables the real-time calculation of the sensor's resistance value, followed by determining the actual monitored quantity based on the pre-established correspondence between resistance values and monitoring quantities. Therefore, establishing the precise relationship between the sensor's resistance and the monitored quantity is critical.

In our study, we employed commercially available temperature and RH sensors. These sensors inherently establish a correspondence between the monitored quantity and resistance during the manufacturing process. To ensure sensing accuracy, we calibrated these sensors prior to their actual applications as shown in **fig. S6**.

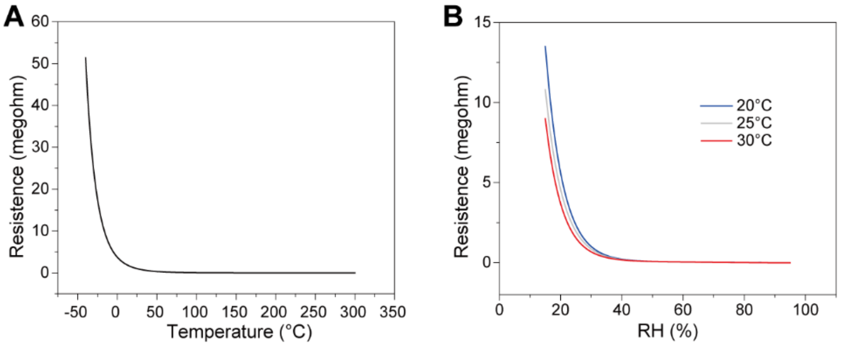

Fig. S6. Resistance characteristics of the commercial temperature and humidity sensors used in this work. (**A**) Temperature-resistance curve of the commercial temperature sensors. (**B**) RH-Resistance curves of the commercial RH sensors.

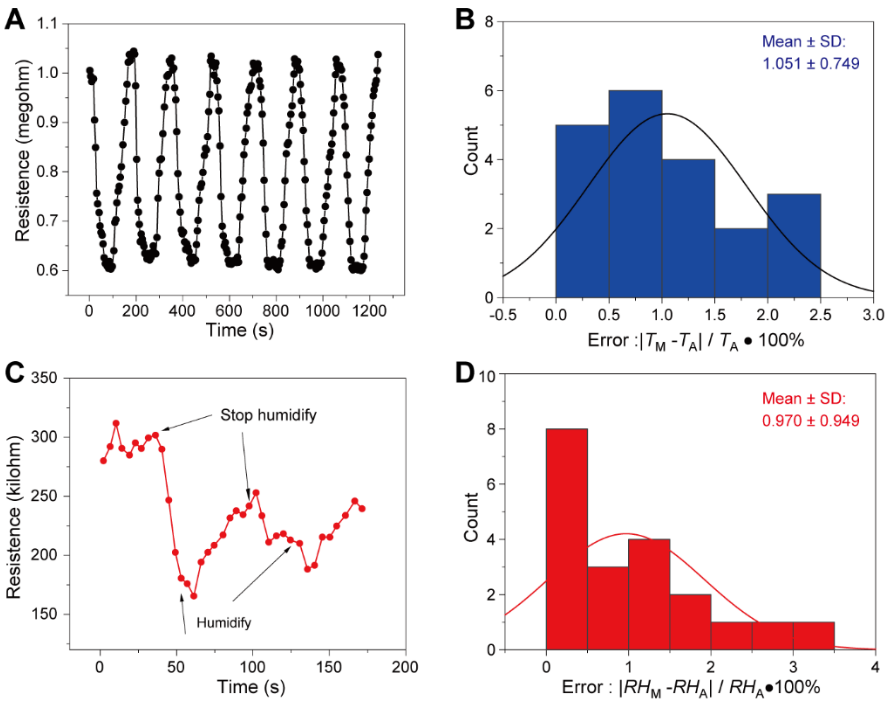

Fig. S7. SSEMS for high-precision temperature and RH monitoring. (**A**) Measured temperature sensor resistance by SSEMS under cyclic test ranged from 25 to 35 °C. (**B**) Errors of 20 temperature sensors powered by SSEMS. (**C**) Measured RH sensor resistance by SSEMS over time under a process of humidify. (**D**) Errors of 20 RH sensors powered by SSEMS.

Supplementary Text 4. **A possible solution to address the limitation of SSEMS only work under rainy conditions**

Regarding the possible limitation of this SSEMS. We considered that sensing performance of system cannot work without raindrops, and tried our best to address this limitation and found a possible solution, that is, integrating complementary energy (solar, wind, etc.) for the self-powered sensing system.

<u>(1) Sensing performance of SSEMS powered by solar cell</u>

The solar cells used here (**fig. S8A**) was purchased from HeYi Co., Ltd., which was integrated by 10 cells, with the size of 8.4 cm × 6.1 cm, and the open-circuit voltage could achieve 6.3 V. Based on the self-calibration strategy, we constructed the SSEMS shown in **fig. S8B**, which utilizes a solar cell to power a temperature sensor (fixed at 30 °C). As illustrated in **fig. S8C**, despite the irregular output of the solar cell under fluctuating light conditions, benefited from the self-calibrated strategy, the sensor resistance can be measured in real-time and consistently remains around 0.75 megohms. So, the SSEMS can be self-powered by the solar cell.

<u>(2) Sensing performance of SSEMS powered by W-TENG</u>

To further diversify the energy sources for the SSEMS, we designed a W-TENG to harvest wind energy. **fig. S9A** shows the schematic diagram of the fabricated W-TENG, which includes two Al foil electrodes at the top and bottom acrylic substrates (here Al foils play as both the positive friction layers and the electrodes). The polytetrafluoroethylene (PTFE) film was fixed at the middle of the device by using two bolts, where two narrow air gaps were created between the PTFE films and the Al films, and the device has the inner dimensions of $10 \times 4 \times 2$ cm^3. The air flows into the gaps of the device and induces the vibration of the PTFE film, which can drive the working of the W-TENG. The output performance of the W-TENG is detailed in **fig. S9B** and **fig. S9C**.

To evaluate the sensing performance of the W-TENG-powered SSEMS, we constructed a sensing system powered by W-TENG (**fig. S9D**), with the temperature sensor fixed at 30 °C. The experimental results (**fig. S9E**) demonstrate that by measuring the current in the calibration resistor branch and the sensor branch, the sensor's resistance value can be calculated in real time and remains stable around 0.75 megohms. This demonstrates that the self-calibration strategy enables the W-TENG-powered SSEMS to function properly, even with fluctuations in the W-TENG output. So, the SSEMS can also be self-powered by the W-TENG.

<u>(3) Sensing performance of SSEMS synergistically powered by R-TENG, solar cell and W-TENG</u>

We systematically investigated the sensing performance of SSEMS by R-TENG, W-TENG, and solar cells, applying it to power a temperature sensor fixed at approximately 36 °C. As shown in **fig. S10**, we designed four powering schemes: (i) R-TENG with W-TENG, (ii) R-TENG with solar cell, (iii) W-TENG with solar cell, and (iv) synergistic powering by R-TENG, W-TENG, and solar cells. The results indicate that, across different powering schemes, the SSEMS can power the temperature sensor to work properly, with the measured temperature values remaining stable around 36 °C and exhibiting a normal distribution.

Furthermore, we applied SSEMS for temperature sensing to evaluate its detection precision. As shown in **fig. S11A**, we tested the SSEMS in a temperature range (20 to 50 °C). It can be found that there is a linear relationship between the measured temperature by SSEMS and the actual temperature. At the same time, an exponential relationship between R_S and temperature was measured by SSEMS, which is consistent with the intrinsic temperature-R_S characteristics of the commercial temperature sensor. Upon further analysis, the detection error (relative error) is less

than 3.0 % (**fig. S11B**). In order to exclude the influence of individual commercial temperature sensors on our evaluation, we tested static self-powered sensing performances (sensing a fixed temperature) of 20 SSEMSs based on 20 commercial temperature sensors around 34 °C, and summarized their sensing performance in **fig. S11C**. As can be seen, the measured temperature by SSEMSs closely matches the actual temperature, and the overall temperature obeys normal distribution (**fig. S11D**).

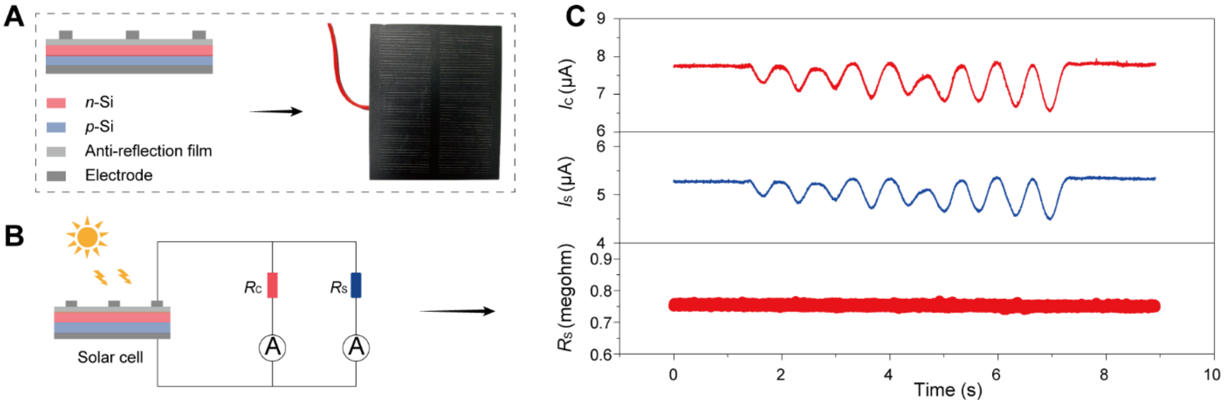

Fig. S8. Sensing performance of SSEMS powered by solar cell. (**A**) Schematic and real image of a solar cell. (**B**) Schematic of a SSEMS powered by solar cell. (**C**) Dynamic R_S (=$R_C \cdot I_C/I_S$) derived from I_S and calibrated by I_C over time in a practical test of SSEMS.

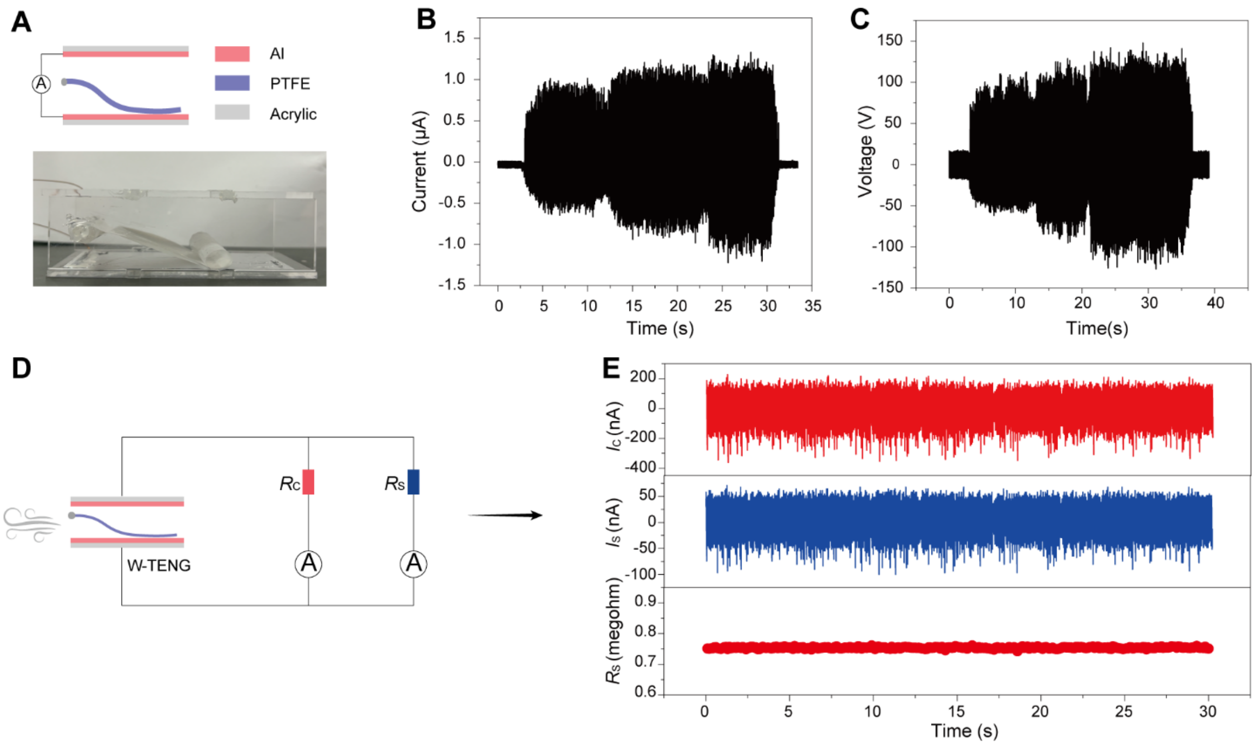

Fig. S9. Sensing performance of SSEMS powered by W-TENG. (**A**) Schematic and real image of W-TENG. Output of current (**B**) and voltage(**C**) of W-TENG. (**D**) Schematic of a SSEMS powered by W-TENG. (**E**) Dynamic R_S ($=R_C \cdot I_C/I_S$) derived from I_S and calibrated by I_C over time in a practical test of SSEMS.

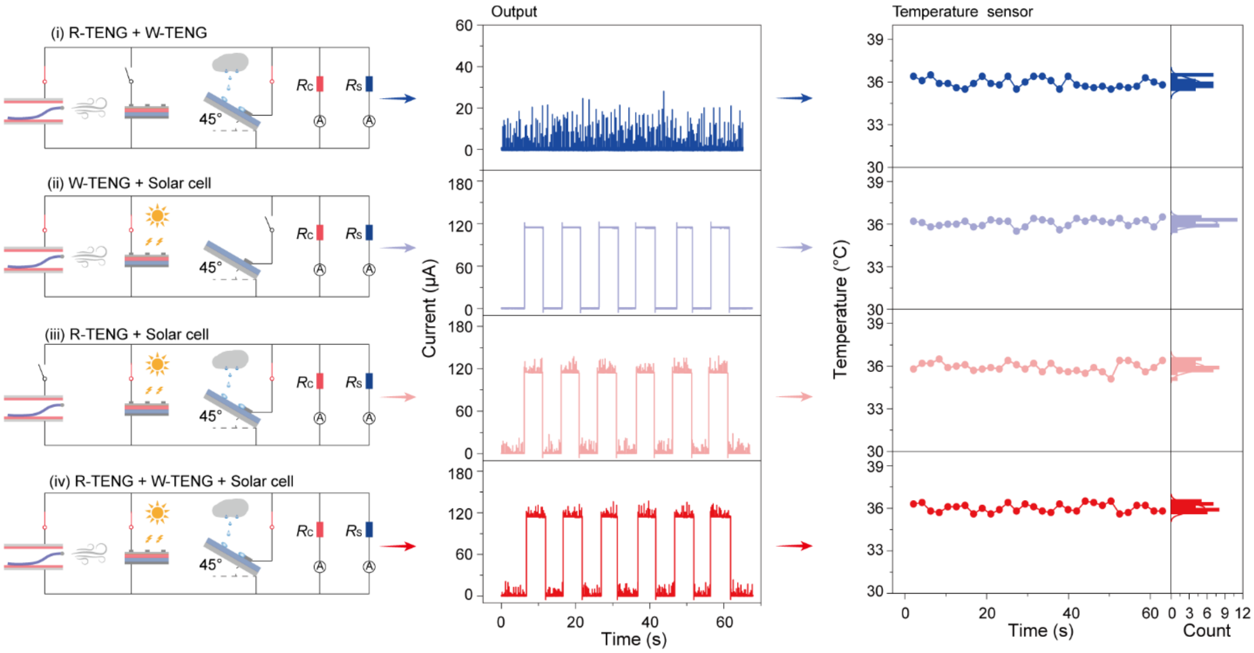

Fig. S10. Real-time sensing performance of SSEMS powered by R-TENG, W-TENG and solar cell synergistically. Sensing performance of the sensing system powered by R-TENG with W-TENG (i), W-TENG with solar cell (ii), R-TENG with solar cell (iii), and R-TENG, W-TENG, solar cell synergistically (iv), respectively. Although different power sources with different output current, the temperature sensing performance of the sensing system is stable.

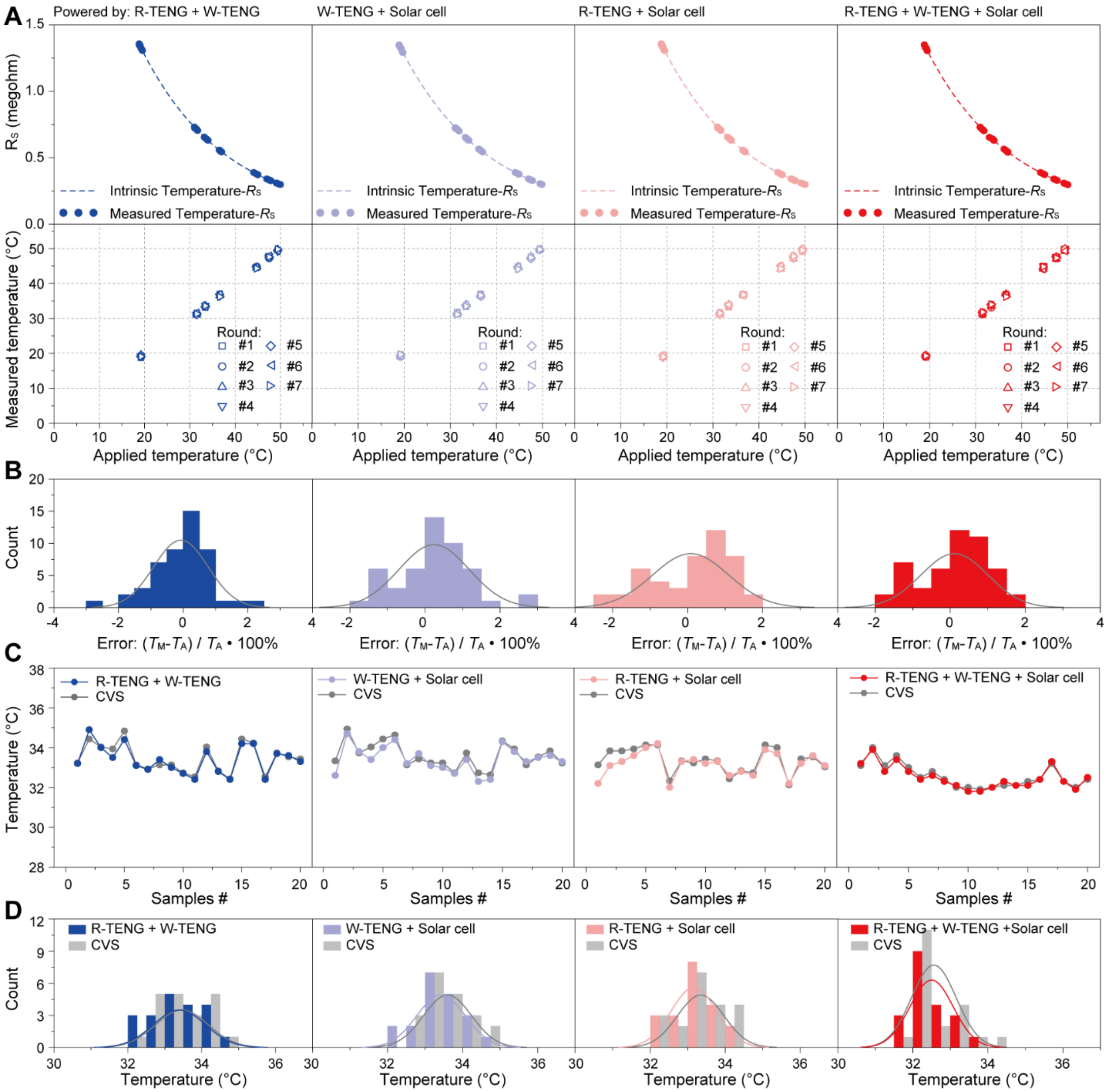

Fig. S11. High-precision temperature sensing of SSEMS synergistically powering by R-TENG, W-TENG and solar cell. (**A**) High-precision static temperature sensing of SSEMS at different temperature, and temperature-*R*s characteristics of a commercial temperature sensor measured by SSEMS (points with different colors) in comparison with the intrinsic characteristics (dotted line). (**B**) Relative error of SSEMS powered by different energy generate devices. (**C**) Summary of static temperature sensing performances of 20 SSEMSs. (**D**) The distribution of temperature values based on (C).

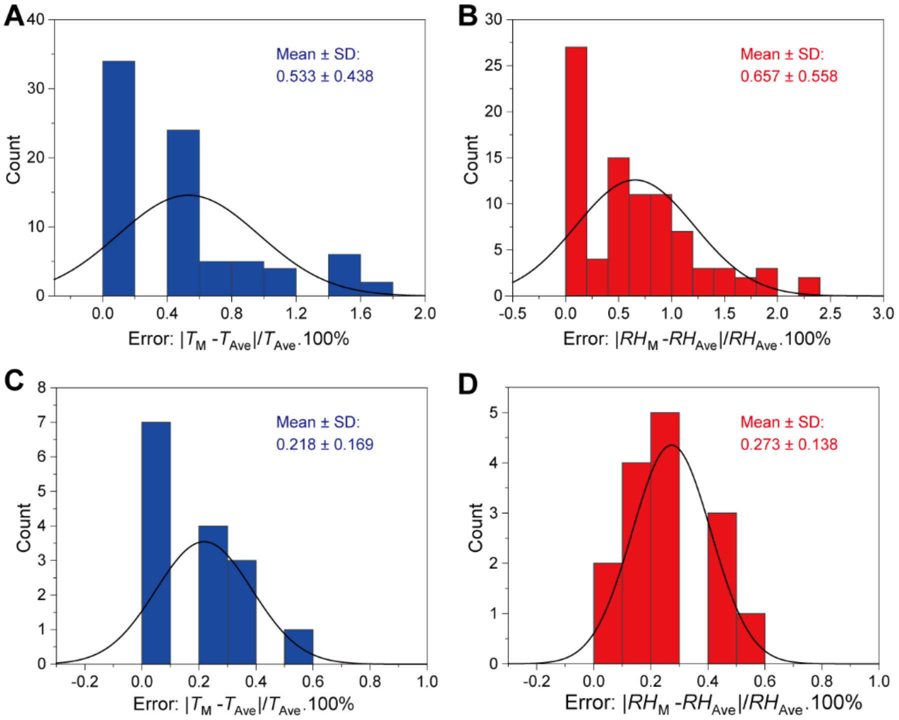

Fig. S12. Relative errors of sensing performance of SSEMS based on R-TENG array for temperature and RH monitoring. (**A**, **B**) Relative errors of the measured temperature (A) and RH (B) calculated from Figure 5d. (**C**, **D**) Relative errors of the measured temperature (C) and RH (D) in SSEMS when powered by different raindrops.

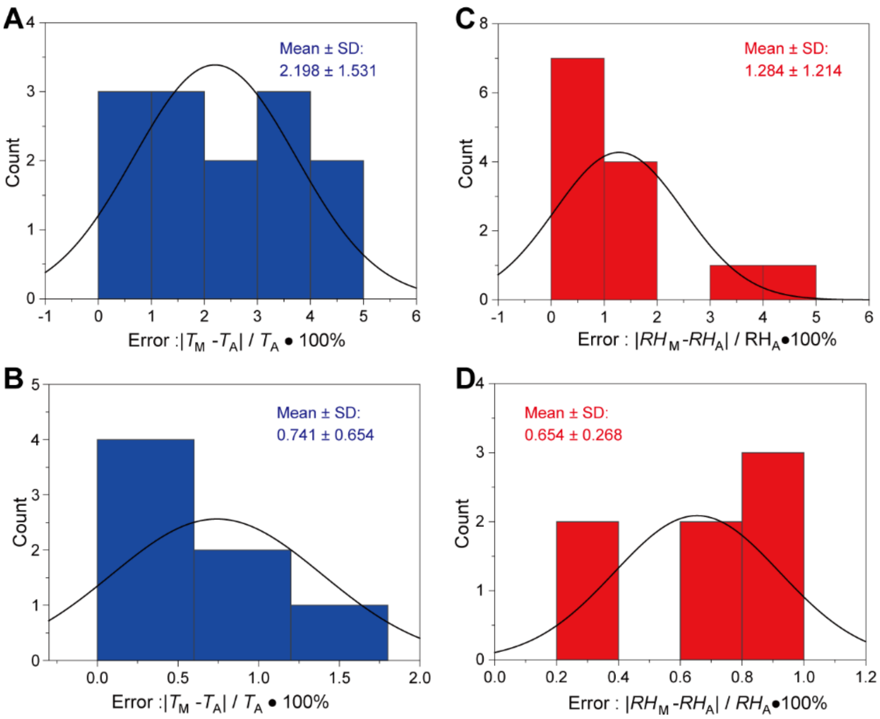

Fig. S13. Relative errors of measured temperature and RH in the practical applications of SSEMS. (**A**, **B**) Relative errors of measured temperature (A) and RH (B) by SSEMS using artificial droplets (waterfalls) within 24 hours at campus of Lanzhou university. (**C**, **D**) Relative errors of measured temperature (C) and RH (D) by SSEMS using natural droplets (raindrops).
